# Centering a pedagogy of care in the pandemic

**DOI:** 10.1177/1473325020981079

**Published:** 2021-03

**Authors:** Gita R Mehrotra

**Affiliations:** School of Social Work, Portland State University, Portland, OR, USA

**Keywords:** Social work education, teaching, pedagogy, reflection

## Abstract

This essay is a reflexive account of my experience of teaching a social justice course during the pandemic. Specifically, I reflect on how centering a pedagogy of care within the course provided a framework for me to be responsive to student needs while also disrupting dominant culture and neoliberal forces in academia. In particular, I highlight sharing power and co-creating meaning, community care, and use of creativity and mindfulness as disruptions to dominant paradigms that I employed in my class that were impactful in the context of the pandemic. I also reflect on how this pedagogical praxis of care has been an instructive and anchoring experience for me as an educator and will impact my teaching going forward.

*To teach in a manner that respects and cares for the souls of our students is essential if we are to provide the necessary conditions where learning can most deeply and intimately begin.* (hooks, 1994: 13)

As COVID-19 was beginning to hit the west coast, the news about our University shifting to remote learning came on the last day of Winter term, giving us a brief window to pivot our courses to online for Spring quarter. Within days, as we continued to learn more about the coronavirus and its impacts, everyone was quickly thrown into a state of collective trauma, grief, and overwhelm. As faculty, we were simultaneously adapting to our new work conditions and needs of students while also trying to make sense of how all of this was going to impact our own lives. Students were confused, overwhelmed, and anxious about remote learning while also trying to manage their personal circumstances, including: having children at home full time, loss of income/jobs, illness, and mental health effects of this pandemic.

In an attempt to support the shift to remote teaching, colleagues and administrators began circulating resources about how to teach in the pandemic and, of course, how to use Zoom effectively. However, even in this initial flurry of communication, messages were conflicting–some narratives emphasized that this was a global pandemic, a social emergency with far- reaching impacts, and that we could not expect that Spring quarter would be “business as usual”. Meanwhile, other narratives underscored the importance of minimal interruption for students and often focused on increasing effectiveness of online teaching. To me, the messages felt inconsistent and reflected tensions inherent to higher education— simultaneously aiming to communicate genuine compassion and care for students in this unprecedented moment, and also operating as a dominant culture, neoliberal institution upholding values of productivity, maintaining the status quo, and a view of students as “consumers” of education. The contradictions between these messages and how they were both upheld and challenged by various players within the institutional space was confusing and, honestly, frustrating.

Despite mixed messages from the institution, it was imperative for me to adapt to the current context and to approach my *Social Justice Practice* class in a way that was congruent with my values. In particular, working from the assumption that the pandemic was a collective trauma, I considered what we know about trauma responses as I developed the course plan. I also knew that given our student population that is diverse in regard to race, class, age, parenting status, and first generation status, students’ lives were likely to be directly affected by the pandemic and I wanted to be as responsive and realistic as possible about what this quarter might look like. Therefore, across all aspects of the course, I aimed to center flexibility, accessibility, and equity.

## Care as a pedagogical anchor

*… people will forget what you said, people will forget what you did, but they will never forget how you made them feel.* –Maya Angelou

While I have always believed in the affective component of education and the relational care needed to have an effective and transformative classroom, during the pandemic it became even more critical to me to center an ethic of care as a pedagogical anchor. I wholeheartedly (and organically) worked from the belief that “demonstrating the act of caring should be one of the ultimate goals of teaching” (Owusu-Ansah and Kyei-Blankson, 2016). In my introductory message to students prior to the start of the term, I sought to reflect this ethos as I wrote:

*Since we have varied levels of experience with online education, different levels of comfort with technology, diverse challenges related to the pandemic and beyond, and varied things going on in our personal lives at the moment, in this space, I ask us to practice self and community care. I ask us to meet each other with flexibility, grace, and compassion as we find our way forward. We will have glitches, we will likely change plans as the course goes on, and we will make mistakes. But, we will also show up, care for one another, read some cool stuff, ask ourselves hard questions about social work and social justice, do our best, and continue to learn and grow. I am committed to doing my best to support you. I care about you and your learning. We are in this together.*

From the start, I reinforced that flexibility, humanity, community care, and personal and family health were our highest priorities for this quarter. It felt vulnerable for me to center this kind of care in the classroom, particularly as a woman of color educator. I especially had concerns about students not taking the course seriously, being seen as a push-over, or being perceived as an ineffective instructor. However, students repeatedly indicated that this approach to the course facilitated their learning during a difficult term, and helped them feel seen and valued as whole people. As noted by one student (via email):

*I want to sincerely thank you for your approach to this term. Your messages share genuine and intimate emotion that I'm sure we all share and you are the only professor (I have 4) to be able to convey the kind of support I have needed to hear. Giving us permission to be lost, scared, frustrated and sad allows me to process stuff that I am forced to hold in when others want to gloss it over with unreasonable positivity. I think my outlook is better now, when I can be ok with feeling bad for a minute.*

To explicitly emphasize an ethic of care as pedagogical praxis felt like a significant disruption to the messaging of the neoliberal institution that we needed to approach teaching/learning as we normally would, and that we could somehow compartmentalize students’ school experience from the larger pandemic context. In my view, student’s learning was already being interrupted in significant ways and centering a pedagogy of care created a space for me to be responsive and promote meaningful learning during a challenging time.

## Sharing power and co-Creating meaning as disruptions to traditional classroom dynamics

Though as educators we often make a clear distinction between our own experiences and that of our students, COVID-19 blurred those lines as we were all part of a collective experience. As such, there were times that I shared my feelings openly with students. I was honest with them about my own challenges with remote teaching, worries about the pandemic and health of my loved ones, and normalized that some weeks were harder to get through than others. These disclosures served to equalize relations of power in the (virtual) classroom in certain ways as students felt like I could understand what they were going through and that we were in a shared experience together.

I also intentionally shared power by working with students to co-create solutions to pandemic-related challenges. For example, when we met via Zoom early in the quarter, it was clear that students were not doing well. In addition to various pandemic-induced life stressors, remote learning was taking a toll on them. Students shared that they were struggling with concentration, tracking information and dates, and keeping up with reading. We collectively discussed options for how we might modify weekly course expectations and, as a group, they made a proposal that they would support one another with reading notes and would take a new approach to the required online discussion. The collaborative culture of the course disrupted the traditional hierarchy between student and instructor as we created shared meaning and adapted aspects of the class together.

## Community care as a disruption to individualism

This class also became a space of deep community care amongst the students which felt like a disruption to the individualism of the neoliberal university and to patriarchal, white, western cultural norms. In addition to strategizing ways to support each other academically, students were authentic and vulnerable in our group check-ins and generously offered mutual aid to one another. Most students attended optional Zoom sessions solely because they wanted to connect with one another. In one session when a student shared that she had been laid off and was stressed about her financial situation, students stepped up and shared campus and community resources (such as food pantries and student emergency funds) with her, and even asked if they could send her money directly. Our last meeting also took place just after the initial Black Lives Matter protests in response to George Floyd’s murder by police. In this session, students openly discussed experiences of the protests, including personal and emotional aspects of being part of this social movement. Several students offered their homes as safe spaces in case of police violence, they shared tips on what to do if exposed to tear gas, and students connected about their lived experiences of the current moment.

The community care that students showed for each other during the term was exceptional, and felt like an extension of the pedagogy of care that permeated throughout the course. It was also an example of praxis, as students were applying with each other what they were learning about in the course: tenets of community work, mutual aid, and anti-oppressive practice. My regular communication with students and various ways that they connected with each other demonstrated the power of relationship in students’ learning, especially in a quarter when many were feeling isolated and disconnected from school and community. When reflecting on the class at the end of the term, multiple students talked about the importance of small group discussions, assignments they completed with a partner, and the learning community we created, as significant to their experience.

## Creativity and mindfulness as disruptions to the “worship of the written word”

Working from the assumption that stress and anxiety were high for everyone, I started each Zoom session with an optional mindfulness and grounding exercise. This was a way to bring students into the space, help calm the nervous system, and share a tool that could be helpful for managing the current trauma(s) of the pandemic. Breathing and grounding together proved a powerful way for students to connect with themselves as a different way of knowing. In addition, I regularly shared quotes and poetry with students in my communications with them to demonstrate creative writing and art as relevant to social change and the circumstances of the pandemic.

“Worship of the written word” is a symptom of white supremacy culture (Okun, 2000) and is central to ways of knowing and learning in higher education. To disrupt this, and to account for what I was hearing from students about reading and writing being particularly difficult in the pandemic, I moved away from papers being the only measure of learning in the course. While I kept two writing-based assignments in the class, I also created a COVID-19 related presentation (focused on the virus and its impacts on communities) and assigned a creative engagement project. The COVID-19 assignment allowed students to learn and present about the current context in relationship to their social work interests. For the creative project, students were asked to do a weekly reflection on the pandemic and/or course content for each week. Students could draw or make art, create a playlist, write a weekly journal or blog post, do a photo-voice project, or suggest an alternative project. Students did a beautiful job with these assignments and their products (including paintings, playlists, homemade jewelry, photographs, zines, and more) demonstrated a deep engagement with course concepts, and authentic meaning-making about various aspects of the global pandemic. Many students shared that the COVID-19 assignment and creative engagement project were the most impactful aspects of the course given their relevance to the moment and the opportunity to practice different ways of knowing and processing information.

## Centering a pedagogy of care beyond the pandemic

*Any classroom that employs a holistic model of learning will also be a place where teachers grow, and are empowered by the process. …most professors must practice being vulnerable in the classroom, being wholly present in mind, body, and spirit.* (hooks, 1994: 21)

There is no question that teaching during the pandemic was a profoundly vulnerable and instructive experience for me as an educator. The emotional labor required to center a pedagogy of care was also significant and, at times, felt overwhelming. While I had grief about the class that we would have had in person and some worry about things that got lost in the shift to remote learning, teaching during this time stretched me as an instructor and person. What I learned most about teaching in this unusual quarter is the importance of staying grounded in my values- namely a pedagogy of care- as a way to be responsive to students, and as a form of resistance to the neoliberal, patriarchal, and white supremacist values that persist in academia. While care is often not explicitly recognized as part of the educational process or as part of our labor as instructors, my experience this term reinforced to me how important care truly is to teaching and learning.

In the last class session, when asked what they would be taking away from the course, one student reflected that one of her biggest learnings- even more than readings or assignments- was the modeling I provided as an educator. Specifically, she named that the way that I approached the course reflected social work values and showed her how she wanted to be as a social worker in the future. In social work education, we often reference the trifecta of knowledge, skills, and values; and yet we don’t always discuss how we teach these values. The way I approached this class helped elucidate social work values for students in ways that I hadn’t expected.

The values that undergirded my teaching in the pandemic were not new, but I was pushed to live into them and trust myself in new ways. This felt especially true as my pedagogy was disrupting “business as usual” and some traditional academic norms and expectations. Throughout the quarter, I often questioned myself as an educator—anxieties about not being tech savvy or experienced with online teaching, concern about the course not being rigorous enough, guilt for struggling to keep up with work, and comparing myself to colleagues who seemed to have shifted to remote teaching with more ease and fewer adjustments. However, it was imperative and values-aligned for me to approach the term and my course in this way, despite messaging from the institution or my own self-doubts. Viewing students (and faculty) as whole people and disrupting the status quo by centering care as a pedagogical anchor required me to be “wholly present in body mind and spirit” and will undoubtedly inform my teaching going forward.
